# Topographic Hub Maps of the Human Structural Neocortical Network

**DOI:** 10.1371/journal.pone.0065511

**Published:** 2013-06-10

**Authors:** Emil H. J. Nijhuis, Anne-Marie van Cappellen van Walsum, David G. Norris

**Affiliations:** 1 Donders Institute for Brain, Cognition and Behaviour, Radboud University Nijmegen, Nijmegen, The Netherlands; 2 MIRA Institute for Biomedical Technology and Technical Medicine, University of Twente, Enschede, The Netherlands; 3 Department of Anatomy, Radboud University Nijmegen Medical Center, Nijmegen, The Netherlands; 4 Erwin L. Hahn Institute for Magnetic Resonance Imaging, University of Duisburg-Essen, Duisburg-Essen, Germany; University of Michigan, United States of America

## Abstract

Hubs within the neocortical structural network determined by graph theoretical analysis play a crucial role in brain function. We mapped neocortical hubs topographically, using a sample population of 63 young adults. Subjects were imaged with high resolution structural and diffusion weighted magnetic resonance imaging techniques. Multiple network configurations were then constructed per subject, using random parcellations to define the nodes and using fibre tractography to determine the connectivity between the nodes. The networks were analysed with graph theoretical measures. Our results give reference maps of hub distribution measured with betweenness centrality and node degree. The loci of the hubs correspond with key areas from known overlapping cognitive networks. Several hubs were asymmetrically organized across hemispheres. Furthermore, females have hubs with higher betweenness centrality and males have hubs with higher node degree. Female networks have higher small-world indices.

## Introduction

Recent studies have investigated the human connectome with graph theory by dividing the neocortex into 100–1000 parcels and examining the anatomical connections derived from diffusion weighted magnetic resonance imaging (DW-MRI or DWI) techniques [1]–[6]. Hubs, highly connected regions, have been of particular interest. They were extensively investigated because of their presumed criticality for the function of the brain [6], [7]. To date it has been shown that neocortical hubs can be found in regions associated with the default mode network [2], [6], [8]. At the same time lesion studies have identified critical brain regions related to important neurocognitive networks [9]–[11]. These critical regions should be considered as candidate hubs, as they are located in highly connected association cortices.

We therefore hypothesized that, besides the default network, other important neurocognitive networks should contain hubs that would be detectable by means of graph theoretical analysis. To test this hypothesis we extend previous work with a detailed map of the neocortex which displays the distribution of its hubs. This is in accordance with previous suggestions to investigate the human connectome in more detail with a larger dataset [6]. Here we present hub maps based on high resolution data, which can be used as a reference for the location of neocortical hubs.

The hubs of a network can be broadly separated into two types: provincial and connector hubs [12]–[14]. Hubs are usually determined with measures which capture the structural importance of a node with respect to the rest of the network [14], [15]. The simplest measure is the degree of a node, which is the number of connections to other nodes and reflects the local importance of a node [16]. Betweenness centrality, which describes the fraction of shortest paths through a specific node, is a good additional measure as it also incorporates global information [16]. We consider it axiomatic that provincial hubs must show high node degree, whereas connector hubs must show a high betweenness centrality. In this paper we used node degree and the betweenness centrality measures to identify hub regions.

To create topographic maps which show how the hubs are distributed, four key aspects were considered in the mapping procedure. First, we used a homogeneous group of 63 young adults with similar age, education and same handedness scanned with a high resolution MRI protocol, which allowed us to make high resolution connectivity matrices. Secondly, we excluded subcortical nuclei from the analysis. The resulting connectivity maps consider exclusively the neocortex and thus avoid mixing polysynaptic with monosynaptic cortico-cortical connections. Thirdly we analysed multiple randomly generated parcellations for each subject in order to have a topographic display of hubs and to minimize node selection biases. And fourthly, to consider the anatomical variability across subjects, we used a surface-based analysis to average the individual maps on a standard brain.

Using our mapping procedure eighteen hub regions on the neocortex were identified which are related to known neurocognitive networks. Furthermore statistically significant differences in the hubs’ distribution across hemispheres and between genders were found.

As differences in hub organization should be related to differences in network topology we complemented our analysis with a small-worldness analysis of the entire brain and for each hemisphere. This approach was chosen, because the small-worldness measure describes global network properties and because the human brain has a small-world topology [7], [17].

## Materials and Methods

### Ethics Statement

The study was conducted at the Donders Institute for Brain, Cognition and Behaviour, Radboud University Nijmegen the Netherlands with the general institutional ethics approval from the local ethics committee (Commissie Mensgebonden Onderzoek region Arnhem-Nijmegen, The Netherlands). All participants provided written informed consent in accordance with the declaration of Helsinki.

### Participants

Sixty-three healthy subjects [37 females, 26 males, mean age, 22.75±2.94 (SD) yr] from the Donders Institute Connectivity Data Set 1 (DICOD1) with 81 subjects under the age of 35 were included for this study. Exclusion criteria for the used dataset were: left-handedness, incomplete DWI data and neurological or psychiatric disorders.

### MRI Acquisition

All subjects were scanned on a Siemens 3T TIM Trio system with a 32 channel head coil at the Donders Institute for Brain, Cognition and Behaviour, Radboud University Nijmegen.

#### Anatomical scan

High resolution anatomical scans were acquired using a T1-weighted 3D MPRAGE sequence with TE = 3.03 ms, TR = 2300 ms, TI = 1100 ms, a flip angle of 8° with 1 mm isotropic voxels.

#### Diffusion weighted imaging

Diffusion weighted imaging volumes were acquired using a single-shot echo-planar imaging (EPI) sequence with phase encoding in the anterior to posterior direction, with TE = 101 ms, TR = 13.0 s, 2 mm isotropic voxels and taken in 256 non-collinear directions at a b-value of 1500 s/mm^2^. In addition, 28 volumes with b = 0 s/mm^2^ were acquired between the diffusion weighted volumes.

### Data Analysis

For each of the 63 subjects twenty different connectomes were generated and estimated for each connectome several network measures.

Before calculating network measures to the neocortical network the nodes and edges need to be defined. While edges are considered to be represented by axonal connections in the subcortical white matter and can be estimated using different DWI techniques, the question of what constitutes a neocortical node is undetermined. Previous work has used fixed anatomical based templates across a population of subjects [1], [4]. Their approach benefits from being able to compare anatomically identical nodes across subjects. Previous work has shown that defining the nodal configuration with anatomical templates may lead to inappropriate node representations which then can lead to incorrect functional network estimates [18] or may poorly characterize U-fibres [19]. We therefore resolved this dilemma using a template free approach and individually parcelled each of our 63 subjects twenty times, in order to reduce the effects of node selection biases and potential fragmentation of hubs.

A detailed description of the processing steps is given in the following sections.

### Step 1: Creation of Neocortical Network Nodes

The anatomical scans were analysed using Freesurfer [20] to segment the brains into cortical and subcortical structures. We used the recommended processing pipeline which included manually correcting for Talairach alignment, skull removal, white matter surface and grey matter surface errors. One subject was excluded from the DICOD1 dataset as grey matter hyperintensities could not be corrected.

Each neocortical hemisphere was then parcelled twenty times into 500 ROIs using the k-means algorithm [21] informed with the Euclidean distances between grey matter voxels. The procedure is not deterministic as the final parcellation dependants on the random initialization of the k-means. The process therefore produced twenty different neocortical parcellation schemes for each brain. The contiguous ROIs of a parcellation had an average size of 0.1% ±0.016% (SD) of the total neocortical volume. Each ROI then defined a node in the structural connectivity mapping step.

### Step 2: Diffusion Preprocessing and Tractography

The diffusion-weighted images were checked for motion, cardiac and table vibration-induced artefacts using the PATCH algorithm [22]. The volumes were then realigned and corrected for eddy current-induced distortions with the integrated approach described in [23]. Finally the volumes were unwarped in the phase encoding direction onto the anatomical scan to reduce the effects of phase evolution in the EPI read out direction [24]. We used the multi-fibre reconstruction PASMRI with 16 basis functions [25] and performed interpolated deterministic tractography using Euler’s algorithm with a 0.2 mm step size seeding on the 1 mm isotropic voxels of the coregistered Freesurfer white matter mask with a maximum of three main principal diffusion directions. The choice of the reconstruction and tractography methods was driven by results presented in [26], who showed that using a spherical deconvolution transform reconstruction in combination with deterministic tractography results in the highest fraction of valid fiber tracts found in a phantom.

### Step 3: Structural Connectivity Mapping

For each brain a network was then reconstructed by defining the ROIs as nodes and the number of tracked fibres between ROIs as the edge strengths. The network matrices were then binarized without thresholding the strength of a connection. The appearance of the hub maps did not substantially change when thresholding, therefore we opted against it as any threshold would have been arbitrarily chosen. The connectivity matrices, with an average of 9.77%±1.03%(SD) of all possible connections, were then used for graph theoretical analysis.

### Step 4: Connectome Analysis

**Figure 1:**
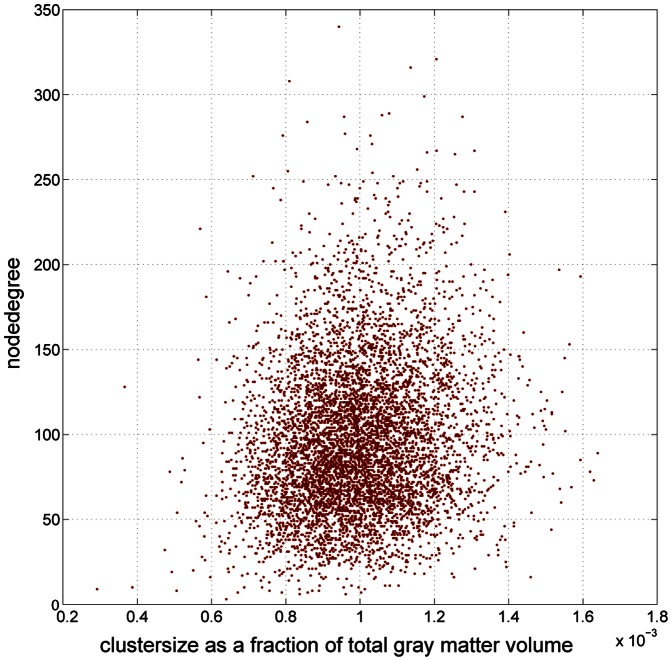
Scatter plot between node degree and cluster volume size. Scatter plot describing the relationship between node degree [mean 97.59±43.73(SD)] and cluster volume size as a fraction of the entire grey matter volume [median 0.099%, 0.016% (SD)] of a subset of 6,300 brain network nodes from all subjects. The correlation of the measures between all nodes is r_1259998_ = .15.

A network analysis was performed with the Brain Connectivity Toolbox [27] to determine the node degree and betweenness centrality for all twenty parcellations of each brain. A correlation analysis (see Figure 1) on the node degree and cluster size of all nodes across all brains concluded that the node degree could not be predicted from the cluster size as r_1259998_^2^ = .023. Therefore it can be assumed that the network measures calculated for each node do not reflect a cluster size dependent artifact induced by the parcellation heuristic.

To compute subject specific hub maps each voxel’s degree and betweenness centrality was taken as an average of the twenty clusters which it fell within. The results were then projected from voxelspace onto the cortical surfaces using Freesurfer.

### Step 5: Mapping Network Parameters to Average Surface

**Figure 2:**
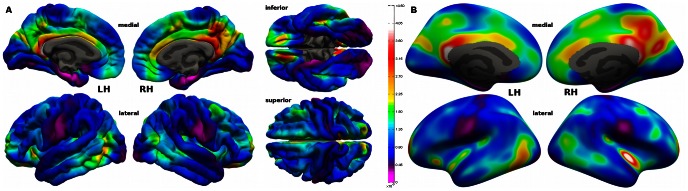
Betweenness centrality hub map. Average betweenness centrality pial (A) and inflated (B) surface hub map with a mean betweenness centrality of 0.00124±0.00061 (SD). The colour scale for the betweenness centrality values is shown at the right of subfigure (A). See also Table S1.

**Figure 3:**
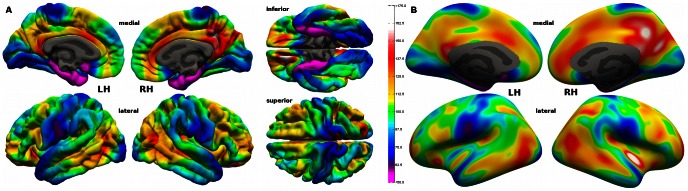
Node degree hub map. Average node degree pial (A) and inflated (B) surface hub map with a mean node degree of 102.57±19.78 (SD). The colour scale for the node degree values is shown at the right of subfigure (A). See also Table S1.

In the last steps we registered all subject specific maps to the Freesurfer average surface, to overlay anatomically identical areas. They were then smoothed with a 10 mm full width at half maximum kernel, to decrease spatial variability between subjects of putative hub areas. Finally the individual hub maps were averaged leading to topographic hub maps, displayed in Figures 2 and 3.

### Step 6: Identifying Hub Regions and Hub Score Asymmetry/Gender Analysis

**Figure 4:**
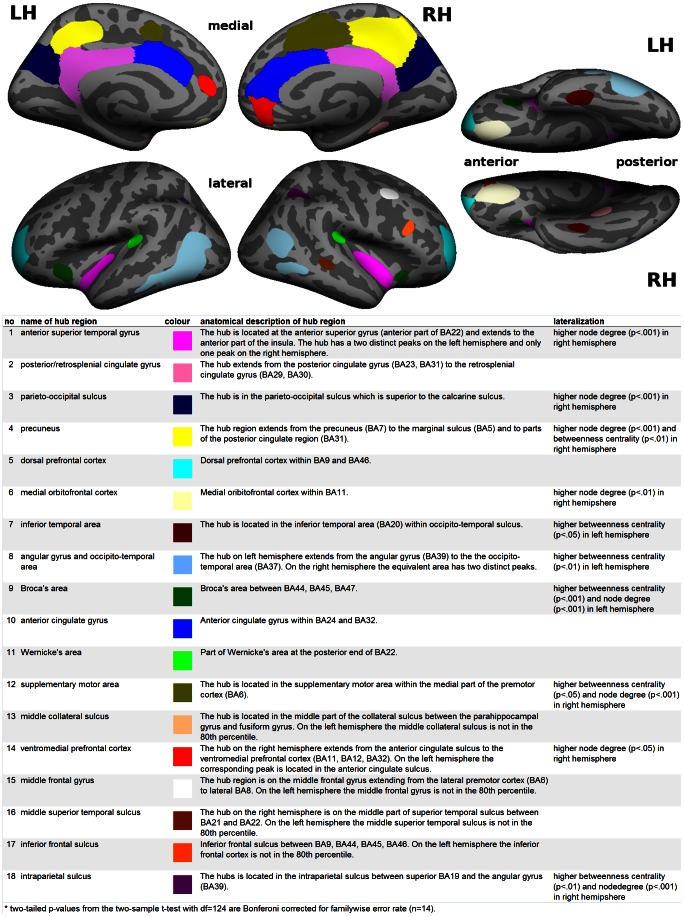
Hub regions with betweenness centrality scores in the 80^th^ percentile displayed on inflated brain surfaces. The anatomical descriptions and lateralization patterns of the coloured hub regions are given in the bottom table. See also Table S2.

Regions on the topographic betweenness centrality map with values in the 80^th^ percentile were defined as hub regions (see Figure 4). This definition led to large contiguous hub regions in the medial cortices which encompassed several independent peaks. Using Freesurfer ROI drawing tools we then defined regions of interest by separating areas along the inflection points between distinct peaks. For the left anterior superior temporal gyrus and the right inferior parietal lobe/posterior temporal lobe/anterior occipital lobe region two distinct peaks were combined to match the corresponding contra-lateral areas. We then defined the maximum value in node degree or betweenness centrality within a region as its hub score. The regions were also used to determine hub scores for individual subjects. Using a two-sample t-test we then tested for statistically significant differences between the hub scores from individuals of anatomically corresponding regions across hemispheres. We also used the same procedure to test for gender differences in the hub scores.

### Step 7: Calculating and Analysing Small-world Indices

For each brain small-world indices σ were calculated for the entire connectivity matrix, the subgraph representing the left hemisphere σ_lh_, the right hemisphere σ_rh_ as well as the difference δ_lh−rh_ = σ_lh_−σ_rh_ of both hemispheres, which we defined as the small-world asymmetry index. Small-world indices were estimated by calculating the fraction σ in equation 1 which is determined by the average cluster coefficients *C* and *C*_rand_ of all nodes and the characteristic paths γ and γ_rand_ in a network and an equivalent random constructed network [28].
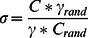
(1)


As each brain was parcelled twenty times the average of each index across the parcellations was used. We analysed the interaction of the small-world indices with each other, with gender and brain volume, as computed by Freesurfer, using SPSS.

## Results

We analysed the neocortical connectomes of 63 young adults extracted from a multi-modal MRI dataset and mapped the outcomes on the Freesurfer [20] group average brain. In Figures 2 and 3 the topographic hub maps are displayed on the Freesurfer standard brain surface, resulting from averaging betweenness centrality and the node degree values across subjects.

### How Hub Regions were Defined

By comparing the node degree and the betweenness centrality maps we observed that in the human brain hub regions are more pronounced in the betweenness centrality map. This can best be explained with regard to the distributions of the hub values. The distribution of betweenness centrality values for individuals appear to follow a power law with a long tail whereas node degree values appear normally distributed skewed to the right with a longer tail. The appearance of the node degree distribution classifies the produced networks as single-scale small-world networks [29]. To define hub regions we used the betweenness centrality map, because the distribution of betweenness centrality values had a longer tail than the distribution of node degree values. Following the Pareto principle [30], we used the 80^th^ percentile of the betweenness centrality map (vertices with values above 0.00164) to define hub regions of interest (see Materials and Methods step 6).

### Anatomical Locations of Hub Regions

We identified eighteen hub regions based on the topographic betweenness centrality hub map (see Figure 4). A description of the anatomical locations can be found in the table of Figure 4. Neighbouring hub regions were manually separated at their inflection line. For readability we will sometimes refer to hubs with their numbers from the table in Figure 4 written in brackets.

In all but four cases we found bilateral hub representations. For hub (1) the anterior lateral sulcus in the left hemisphere and hub (8) in the angular gyrus and occipito-temporal area, we combined two distinct peaks to a single hub region in order to match the contra-lateral hub region.

### The Hub Scores, their Asymmetry and their Gender Differences

For all but one hub region we could identify a distinctive peak in the node degree and betweenness centrality hub map. The left supplementary motor area (12) in the node degree map did not have a distinct peak and was merged into hub (10). The peak values for all hub regions on the average brain are listed in Table S1.

Every pair of bilateral hub regions was tested for asymmetry by comparing across hemispheres the maximum individual values for each hub region (see Materials and Methods step 6). With the asymmetry analysis we found statistically significant differences between corresponding hub regions across hemispheres (see Figure 4 for the outcomes and Table S2 for full results). The results were Bonferroni corrected (n = 14) to account for familywise error rates. Wernicke’s area (11) was the only region with a reverse lateralization pattern for the node degree and betweenness centrality hub scores. However neither lateralization was statistically significant. All other regions showed a consistent lateralization for both node degree and betweenness centrality. In total six hub regions showed statistically significant hemispheric differences in their betweenness centrality scores and eight hub regions had statistically significant hemispheric differences in their node degree scores. For four regions both node degree and betweenness centrality scores were statistically significant lateralized.

Comparing the hub scores between genders (see Table 1) shows that the node degree scores for the male are higher for all but two hubs (p_FWER_(X≤2)<10^−6^). The betweenness centrality hub scores on the other hand were higher for females in 24 out of a possible 32 regions (p_FWER_(X≤8)<.01). The p-values are derived by considering that the inequalities in Table 1 should have been binomially distributed in absence of gender differences, including Bonferroni correction (n = 2) for familywise error rates.

**Table 1:** Gender differences of hub scores.

		betweenness centrality		nodedegree	
id	name of hub region	left hemisphere	right hemisphere	left hemisphere	right hemisphere
1	anterior superior temporal gyrus	F>M*	F>M	F>M	M>F
2	posterior/retrosplenial cingulate gyrus	F>M	F>M	F>M	M>F
3	parieto-occipital sulcus	M>F	F>M	M>F* †	M>F**†
4	precuneus	F>M*	F>M	M>F	M>F*
5	dorsal prefrontal cortex	M>F	M>F	M>F**†	M>F* †
6	medial orbitofrontal cortex	F>M	M>F	M>F	M>F*
7	inferior temporal area	F>M	F>M	M>F	M>F*
8	angular gyrus and occipito-temporal area	F>M	F>M	M>F	M>F
9	Broca’s area	F>M	F>M	M>F**†	M>F
10	anterior cingulate gyrus	F>M	F>M	M>F	M>F
11	Wernicke’s area	F>M	F>M	M>F	M>F
12	supplementary motor area	F>M	F>M	M>F	M>F
13	middle collateral sulcus	n.a.	F>M	n.a.	M>F
14	ventromedial prefrontal cortex	M>F	F>M	M>F*	M>F* †
15	middle frontal gyrus	n.a.	F>M	n.a.	M>F
16	middle superior temporal sulcus	n.a.	M>F	n.a.	M>F**†
17	inferior frontal sulcus	n.a.	M>F	n.a.	M>F
18	intraparietal sulcus	F>M	M>F	M>F* †	M>F***†

F>M marks that the average female hub score was larger than the male average hub score, while M>F marks the opposite.

* , ** and *** mark that the regions’ hub scores differed statistically significantly without corrections for multiple comparisons between genders with t_61_>|2.00|, p_2-tailed_<.05; t_61_>|2.66|, p_2-tailed_<.01 and t_61_>|3.46|, p_2-tailed_<.001 respectively.

† mark that the regions’ hub scores differed statistically significantly between genders with false-discovery rate adjusted (q = 0.05, n = 32) p-values of p_2-tailed,FDR_<.05.

At the single hub level we also performed independent two sample t-tests to determine which hubs differed most between genders. Thirteen out of 32 bilateral hubs were statistically significantly different between genders, without correction for familywise error rates. Nine hubs differed statistically significantly between genders when adjusting the p-values for false discovery rates (q = 0.05, n = 32) [31]. This gives a strong indication that hub scores in general differ between genders, although a larger sample size is needed to more specifically identify the hubs concerned.

### Small-world Network Analysis

For the hub areas most node degree and betweenness centrality scores in the right hemisphere are higher than in the left hemisphere. This result, together with the observed gender differences, indicates that gender differences in the network topologies may exist. To have a more complete understanding of the asymmetry and gender differences in the neocortical network we performed a small-world network analysis (see Materials and Methods step 7).

**Figure 5:**
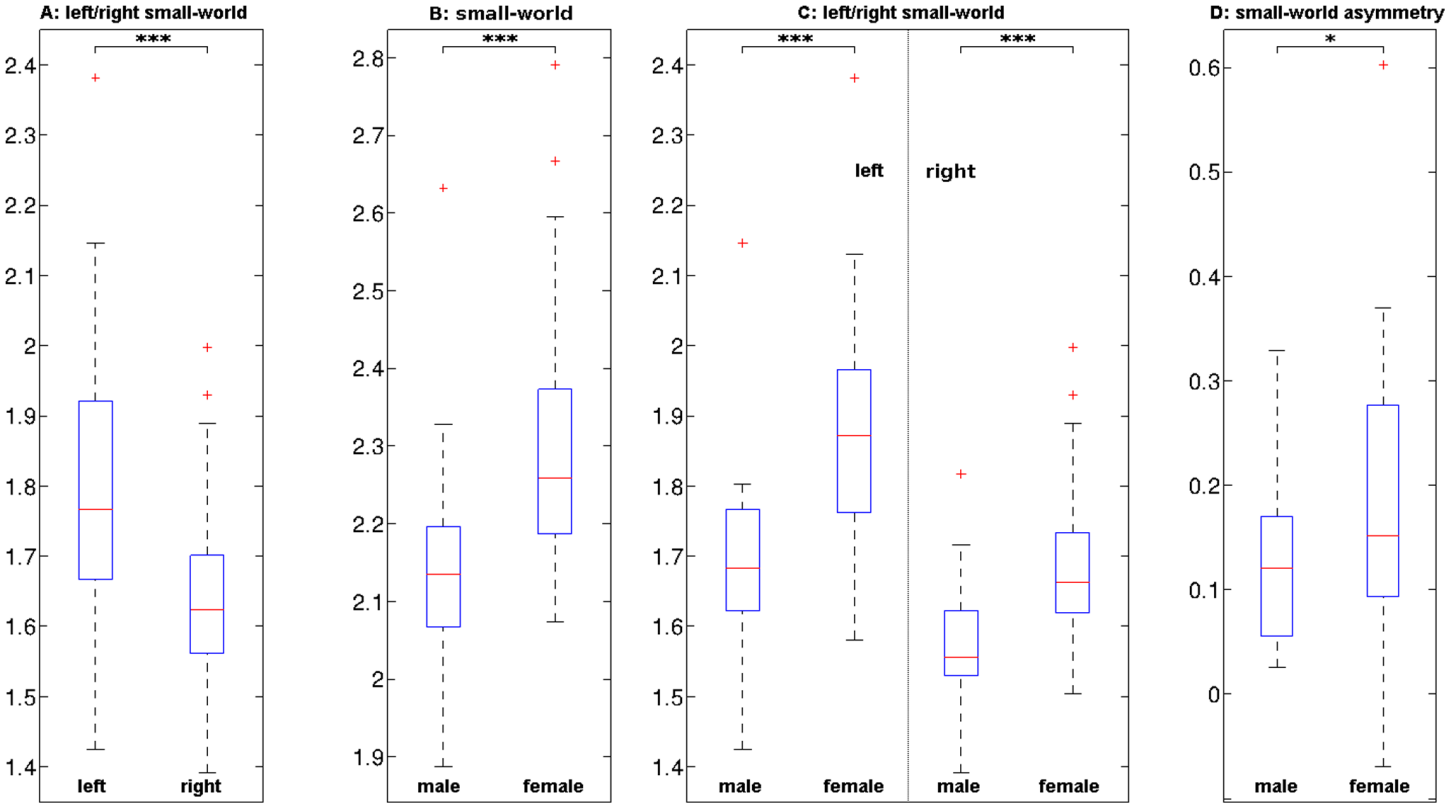
Gender and hemispheric differences in small world indices. The differences between left and right hemispheric small-world indices are shown in boxplot (A). Boxplots grouped by gender are: (B) whole brain small world indices, (C) left and right hemispheric small world indices and (D) small world asymmetry indices. See also Tables S3 and S4. Figure 5 footnote: *** and * indicate statistical significant differences with p_2-tailed_<.001 and with p_2-tailed_<.05. The degrees of freedom for the tests are A: df = 124, B-D: df = 61. Each boxplot shows the median (red line), the upper and lower quartile (blue rectangle), the smallest and largest observations (endpoints of the dashed line) and observations which should be considered as outliers (red pluses).

The results of the whole brain small-world index analysis are displayed in the boxplots of Figure 5 (for mean values and standard deviations see Tables S3 and S4). A correlation analysis between the left and right small-world indices found that these are related with r_61_ = .76, while a paired t-test revealed that the left hemisphere has a statistically significant higher (t_124_ = 6.09, p_2-tailed_<.001) small-world index. Using an independent two sample t-test we found statistically significant gender differences (p_2-tailed_<.001) for the whole brain (t_61_ = 3.61), left (t_61_ = 4.46), and right (t_61_ = 4.47) small-world indices. The difference between the small-world indices of each hemisphere was statistically significantly different between genders with (t_61_ = 2.01, p_2-tailed_<.05). Since the female brain tends to have a smaller volume than the male brain it was plausible that the gender differences in the small-world indices are related to difference in brain volume. Correcting for brain volume with an analysis of covariance showed that brain volume is not a confounding factor for the gender differences. Small-world indices were in fact uncorrelated with grey matter volume (r_61_ = −.31), with white matter volume (r_61_ = −.31) and with the combined grey and white matter volume (r_61_ = −.33).

## Discussion

This study shows the distribution of provincial and connector hubs in the healthy human brain. In connection with a network analysis, general conclusions may be drawn regarding the lateralization of the major networks, and gender differences in network structure. These results and their implications will be discussed below after we have addressed some of the methodological considerations associated with the study.

### Methodological Considerations

A common error source in brain network modelling is the selection of nodes [19]. It has been shown that contemporary structural atlases provide inappropriate node definitions [18]. By using twenty random parcellations over a single subject we were able to obtain a topographic display of hub regions independent of predefined anatomical boundaries. This template free network reconstruction approach proved to be beneficial, as hub regions were found at the boundaries of classical anatomical areas.

The discovery of false white matter connections is a well established problem in studies using fibre tractography. With no existing gold standard techniques or histological based fibre atlases of the whole human brain, fibre tracts cannot be validated for individual brains. We minimized this potential source for errors by using a sophisticated multi-fibre reconstruction method from the Camino package [25] on high angular resolution diffusion imaging (HARDI) data [32] and informed our tractography using the Freesurfer grey and white matter segmentation routines.

Some computed callosal fibre tracts appeared to terminate in the cingulate cortex and while other studies have similar findings [33], [34] it is possible that their existence is rooted in the limitations of current available data and processing software, as they are a likely artefact of partial volume voxels [35]. Reducing errors in the tractography will lead to improved accuracy of hub scores due to reduction of partial volume effects, specifically in regions connected with callosal and uncinate fasicle fibre pathways [35], [36].

### The Location and Ranking of the Hubs

Hubs were located on both maps in comparable regions, however the betweenness centrality had more pronounced hub regions than the node degree map. This observation was consistent with our assumption that betweenness centrality is a marker for connector hubs. The betweenness centrality map was therefore used to determine important hub loci on the neocortex.

We identified eighteen peaks reflecting distinctive hub regions. The ranking and location of the hubs shows correspondences with previous literature using structural connectivity analysis [2], [4] and partially overlap with hubs determined by network analysis of resting-state functional MRI data [37]–[39]. Because of the topographic approach we discovered new spatial detail in the distribution of the hubs. For instance we found three distinct hubs in the posterior cingulate cortex and medial parietal lobe, while previous findings [2], [4] suggested a single hub region in the same area. With the topographic maps some new hub areas become recognizable, such as the inferior temporal area (7), Broca’s area (9), the supplementary motor area (12), and the middle frontal gyrus (15). The new hubs are also known to be key areas in neurocognitive networks [40]–[43].

The superior temporal gyrus was the hub with the highest peak value for betweenness centrality. This result may be surprising as previous literature considers the medial parietal lobe at the core of the neocortical structural network [2], [4]. However a higher peak hub value on an average topographic map does not necessarily imply a higher importance, but could be caused by anatomical variability between subjects. For example we observed that the hub in the left superior temporal gyrus (1) had for each subject either an anterior peak or a slightly more posterior peak. Hence the average betweenness centrality hub map showed two peaks, which we assume to belong to the same hub region. The length of the left lateral sulcus is known to be longer than its contralateral homologue, which in turn explains the asymmetric appearance of hub (1). Furthermore as will be discussed below, the medial parietal hub of previous papers is here differentiated into three separate hubs.

Instead of focusing on the precise ranking of the hubs, we will focus in the following paragraphs on the functional roles and the asymmetry patterns of the hubs matching the results to known neurocognitive networks from previous literature. The hubs cover a broad range of functions, but for simplicity we chose to discuss them in the context of four specific networks. All but one hub can be associated with the default mode network, visual processing networks or networks related to language processing. Many of the hubs can be associated to more than one of those networks. The hubs will therefore be discussed in the context of all three of these networks. The only exception is hub (17) in the right inferior frontal cortex, which is an area associated with the cognitive control network and has been indicated to be an important area for making risk-taking and go/no-go decisions [44]–[47].

### The Hubs Related to the Default Mode Network

The largest fraction of hubs can be anatomically linked to the default mode network, a set of neocortical regions which is active during rest [8]. The regions related to the default mode network can be identified using different types of fMRI analysis [48]. In total we found thirteen hub regions (2–10, 13, 14, 16, 18) overlapping with the default mode network defined in previous literature [8], [48]–[51].

While studies using functional MRI (fMRI) show differences in what encompasses the default mode network, all consider the posterior medial parietal lobe to be integral to its functioning. Previous studies focusing on the topology of the structural neocortical network found that the precuneus and the posterior cingulate cortex form a hub region [2], [4].

The topographic map separated the medial parietal lobe and the posterior cingulate cortex into three hub regions: the posterior/retrosplenial cingulate gyrus (2), the parieto-occipital sulcus (3) and the precuneus (4). This suggests that the posterior part of the default mode network can be further subdivided in three subnetworks. Some recent fMRI studies have subdivided the medial parietal lobe on the basis of functional connectivity patterns and found corresponding results [52], [53]. As the default mode network involves a large area around the medial parietal lobe, there is a considerable anatomical overlap with other neurocognitive networks, such as the spatial awareness, working memory and executive function networks [8], [10]. The precuneus part of the default mode network has been found to overlap with executive activity [54], whereas the parietal-occipital sulcus can be related to working memory tasks involving visual input [55]. The three distinctive hub regions could therefore reflect distinctive functional roles of each of the regions.

Considering the lateralization of the hubs in the medial parietal lobe we found that the precuneus (4) and the parietal occipital sulcus (3) had statistically significantly higher node degree scores in the right hemisphere than their contra-lateral homologues. This finding is also consistent with [4], [6] who identified similar characteristics when using large scale neocortical nodes. However we did not find statistically significant differences between hemispheres for the posterior and retrosplenial cingulate gyrus (2).

### The Hubs in Relation to the Visual Processing Networks

Six hubs (1, 3, 8, 12, 15, 18) can be linked to different networks involving visual processing, such as the network of spatial awareness [10], [56], visual attention network [57] and networks related to visuo-motor coordination and execution [49].

Hubs (3, 12, 15, 18) are anatomically associated with the spatial awareness network, which is lateralized to the right hemisphere [10], [58]. At the same time the related hubs to the spatial awareness network are lateralized to the right hemisphere, consistent with literature which considers that damage to the right hemisphere causes more severe neglect [59].

The hub in the parieto-occipital sulcus (3) links areas in the occipital lobe and in the parietal lobe and is hypothesized to play an important blocking role in the dorsal information flow from visual areas [55]. Furthermore hub (3) is considered to be part of the network for working memory and executive function [10].

Hub (8) can also be associated with both the default mode and language networks. However posterior areas of hub (8) also coincide with associative visual cortex, specifically the subregion TO2 which is part of the MT+ complex (motion-selective cortex) [60], [61]. Considering the partial overlap with various known neurocognitive networks it is possible that hub (8) is a composite of multiple spatially distinct hubs which are combined because of individual anatomical variability, spatial resolution and the smoothing kernel used. This assumption is strengthened by two distinct peaks on the right hemisphere in the equivalent region.

### The Hubs Related to the Language Network

Nine hubs (1, 2, 5, 7–9, 13, 14, 16) can be anatomically associated with the language network [11], [62], [63]. Six of these hubs in the left hemisphere (1, 5, 8, 9, 11, 16) are critical for auditory sentence comprehension [11].

The hub in anterior superior temporal gyrus (1) encompasses an area important for voice recognition [64]. Besides the aforementioned anatomical asymmetry of the lateral sulcus, there are also functional hemispheric differences of the anterior superior temporal gyrus related to the emotional processing of voices [65], which may be related to the statistically significant higher node degree in the right hemisphere.

Areas which had higher betweenness centrality hub scores were: the temporal pole (7), the posterior middle temporal gyrus (8) and Broca’s area (9), which is consistent with previous literature which considers that the production of language dominates in the left hemisphere rather than the right [11], [41], [63]. However, unexpectedly Wernicke’s (11) area did not show a leftward asymmetry pattern. This may be explained by the importance of the right posterior lateral sulcus for other cognitive processes, such as activities related to music [66]. This view is further supported by lesion studies which find a region around hub (11) to be critical in the right hemisphere [67]. The aforementioned asymmetry in the scope of hub (8) may also be related to hemispheric differences in language production. In the left hemisphere the inferior part of hub (8) stretches towards the fusiform gyrus, a region which is related with the visual word form area [68], [69].

### Asymmetry of the Hubs and the Topology of the Neocortical Network

There is known lateralization of brain function for language and visuo-motor processes, as well as anatomical brain asymmetries [70]. Recent studies focusing on white matter connectivity have also shown that there are measurable structural hemispheric differences in the superior longitudinal fasciculus and the cingulum, two major fiber pathways in the human brain [71]–[73]. We therefore expected to measure hemispheric asymmetries in the neocortical network.

Seven hubs had significantly higher node degree scores on the right hemisphere compared to only one hub on the left hemisphere. For the betweenness centrality scores each hemisphere had three hubs which were statistically significantly higher than in the other hemisphere. This indicates that the hubs on the left hemisphere are connected with less brain regions than those on the right hemisphere, while each hemisphere has a set of distinctive hubs with high betweenness centrality. The left hemisphere however has higher small-world indices compared to the right hemisphere. Higher small-world indices imply a more efficient network structure for message passing [74], [75].

### Gender Network Differences

With the high resolution connectome analysis we observed several gender differences, which all indicate that the female brain has a higher network efficiency. The results are therefore consistent with previous findings, achieved at a coarser resolution [76].

For the female brain we found that most hubs have higher betweenness centrality compared with hubs in the male brain. On the other hand male brains tend to have hubs with higher node degree compared to the female brain. This shows that female hubs are more economical in the use of connections, while at the same time being more important in their role as connectors. While there is a pattern of gender differences in hub scores, a larger sample size is still needed to more specifically identify the hubs which differ most between genders.

The results of the small-world network analysis between genders were consistent with the observed hub differences. Female brains had higher small-world indices for the whole brain and both hemispheres. This indicates that the female brain, while being smaller in volume and having overall fewer connections in hub regions, has a more effective network structure for message passing [74], [75]. The small-world asymmetry index was found to be statistically significantly higher for females than for the males.

Lesion simulation studies have concluded that the targeted removal of connector hubs or regions with highest betweenness centrality causes the most severe and widespread disruption within the neocortical network [7], [17]. This suggests that most female hubs are more critical to their neocortical network than their male counterparts, since their betweenness centrality scores are overall higher. Therefore it is plausible that a network disruption in a female brain is more severe than in males, because the female brain has a more economical network structure than males while at the same time their hub areas have a more critical role. The gender differences identified in this study therefore may have important implications for studies considering brain injury and disease. For instance clinical studies have found that female are more at risk to have post-stroke disability and have a higher mortality rate after most types of strokes [77]–[82]. So far, it is undetermined what causes gender differences in stroke impact. Pre-stroke disability, sociodemographic factors and hormone exposure are currently among the possible candidates to explain the sex differences [78], [79]. Evidence has suggested that lesion volume is not the cause of gender disparities in stroke outcomes [83].

### Future Work

With the topographic display of hubs, the scope and lateralization of important brain areas became discernible. Our hypothesis was confirmed that with graph theoretical analysis hubs can be found in important neurocognitive networks, besides the default network.

Future studies may benefit from the maps, because they can be used as a reference and new hypotheses regarding the neocortical hubs can be formulated. To extend this work, several other avenues of research can be considered which cover a broad spectrum of topics.

The presented results may be important to studies concerned with brain disease and injury. This is especially true for diseases with focal pathology such as stroke and tumours, but is also relevant for diseases with more global pathology such as Alzheimer’s or Parkinson disease. For instance damage to the hubs after stroke may play an important role in outcome and rehabilitation [7]. Our results show profound gender differences in the organization of the neocortical network which are consistent with observations in stroke literature. Therefore this study provides grounds to examine the role of complex structural brain characteristics in stroke outcomes. In patients with glioma, it is hypothesised that a widespread change in the strength and spatial organization of brain networks is responsible for cognitive dysfunction [84]. To validate the hypothesis one could examine how changes in functional brain networks relate to changes in structural brain network topology.

Previous work has already shown that the brain measurably changes its functional and structural organization with age [85], [86]. Brain developmental and brain ageing aspects are therefore other promising areas which may further benefit from this study. This could be done by investigating whether and how the distribution of hubs alters with age progression.

It will be interesting to examine hub differences in healthy populations and relate them to behavioural indices or biological markers. Studies have related structural brain properties such as cortical thickness to candidate genes [87]. Since there are gender differences in the neocortical network topology, there is also potential to link genetic information with network topology descriptors such as hub scores or small-world indices.

## Supporting Information

Table S1 Peak values of hub regions on the topographic hub maps.
(DOC)

Table S2 Results of the hub asymmetry analysis. Footnote: *, ** and *** mark that the regions’ hub scores across hemispheres differed statistically significantly after Bonferoni correction for familywise error rate (n = 14) with p_2-tailed,FWER_<.05; p_2-tailed,FWER_<.01 and p_2-tailed,FWER_ <.001 respectively. The degree of freedom for the used two-sample t-tests were df = 124.
(DOC)

Table S3 Results of the network topology analysis. The values in the tables are averages with their standard deviations for the female and male group.
(DOC)

Table S4 Results of the network topology analysis. The values in the tables are averages with their standard deviations for the female and male group.
(DOC)
