# Supplementary material for: Topographic Hub Maps of the Human Structural Neocortical Network
Source: PLoS One. 2013 Jun 10;8(6):e65511. doi: 10.1371/journal.pone.0065511 (PMC3677881; doi:10.1371/journal.pone.0065511)
Supplement: Supplementary file 1 [file pone.0065511.s001.doc]

| id | name of hub region | betweenness centrality left hemisphere | betweenness centrality right hemisphere | nodedegree left hemisphere | nodedegree right hemisphere |
|---|---|---|---|---|---|
| 1 | anterior superior temporal gyrus | 0.00233 | 0.00458 | 120.9 | 178.7 |
| 2 | posterior/retrosplenial cingulate gyrus | 0.00436 | 0.00396 | 152.2 | 155.0 |
| 3 | parieto-occipital sulcus | 0.00234 | 0.00368 | 119.6 | 158.7 |
| 4 | precuneus | 0.00218 | 0.00360 | 122.3 | 164.6 |
| 5 | dorsal prefrontal cortex | 0.00350 | 0.00298 | 163.3 | 149.1 |
| 6 | medial orbitofrontal cortex | 0.00276 | 0.00333 | 146.8 | 167.9 |
| 7 | inferior temporal area | 0.00292 | 0.00187 | <118.4 | 121.4 |
| 8 | angular gyrus and occipito-temporal area | 0.00280 | 0.00205 | 141.3 | 142.9 |
| 9 | Broca's area | 0.00265 | 0.00172 | 149.6 | 135.2 |
| 10 | anterior cingulate gyrus | 0.00236 | 0.00259 | 134.6 | 136.3 |
| 11 | Wernicke's area | 0.00205 | 0.00219 | 128.2 | 139.5 |
| 12 | supplementary motor area | 0.00178 | 0.00213 | no seperate peak | 131.7 |
| 13 | middle collateral sulcus † | <0.00164 | 0.00207 | <118.4 | 132.2 |
| 14 | ventromedial prefrontal cortex | 0.00186 | 0.00198 | <118.4 | 134.1 |
| 15 | middle frontal gyrus † | <0.00164 | 0.00198 | <118.4 | 123.3 |
| 16 | middle superior temporal sulcus † | <0.00164 | 0.00180 | 121.0 | 136.1 |
| 17 | inferior frontal sulcus † | <0.00164 | 0.00178 | 120.1 | 130.5 |
| 18 | intraparietal sulcus | 0.00176 | 0.00178 | 123.1 | 136.1 |
