# Supplementary material for: Topographic Hub Maps of the Human Structural Neocortical Network
Source: PLoS One. 2013 Jun 10;8(6):e65511. doi: 10.1371/journal.pone.0065511 (PMC3677881; doi:10.1371/journal.pone.0065511)
Supplement: Supplementary file 2 [file pone.0065511.s002.doc]

id	name of hub region	betweenness centrality left hemisphere	betweenness centrality right hemisphere	nodedegree left hemisphere	nodedegree right hemisphere
1	anterior superior temporal gyrus	0,00438	0,00541	145,2	192.8***
2	posterior/retrosplenial cingulate gyrus	0,00664	0,00673	178,8	186,8
3	parieto-occipital sulcus	0,00483	0,00588	158,7	191.2***
4	precuneus	0,00402	0.00645**	162,3	202.0***
5	dorsal prefrontal cortex	0,00538	0,00484	190,4	183,5
6	medial orbitofrontal cortex	0,00376	0,00457	165,5	188.8*
7	inferior temporal area	0.00389*	0,00263	137,7	130,1
8	angular gyrus and occipito-temporal area	0.00584**	0,00410	186,3	180,0
9	Broca's area	0.00333***	0,00216	162.6*	145,5
10	anterior cingulate gyrus	0,00376	0,00477	164,1	179,7
11	Wernicke's area	0,00282	0,00250	143,5	148,1
12	supplementary motor area	0,00275	0.00445*	144,2	173.3***
14	ventromedial prefrontal cortex	0,00264	0,00360	138,9	164.0*
18	intraparietal sulcus	0,00196	0.00289**	135,4	166.4***
