# Supplementary material for: Topographic Hub Maps of the Human Structural Neocortical Network
Source: PLoS One. 2013 Jun 10;8(6):e65511. doi: 10.1371/journal.pone.0065511 (PMC3677881; doi:10.1371/journal.pone.0065511)
Supplement: Supplementary file 3 [file pone.0065511.s003.doc]

	small-worldness σ	left hemisphere small-worldness σ_{lh}	right hemisphere small-worldness σ_{rh}	small-worldness asymmetry δ_{lh-rh}
male	2.14 ± 0.14 (SD)	1.70 ± 0.13 (SD)	1.57 ± 0.09 (SD)	0.19 ± 0.13 (SD)
female	2.30 ± 0.16 (SD)	1.87 ± 0.17 (SD)	1.69 ± 0.11 (SD)	0.13 ± 0.09 (SD)
