# Supplementary material for: Topographic Hub Maps of the Human Structural Neocortical Network
Source: PLoS One. 2013 Jun 10;8(6):e65511. doi: 10.1371/journal.pone.0065511 (PMC3677881; doi:10.1371/journal.pone.0065511)
Supplement: Supplementary file 4 [file pone.0065511.s004.doc]

	whole brain		left hemisphere		right hemisphere	
	C/C_{rand}	λ/λ_{rand}	C/C_{rand}	λ/λ_{rand}	C/C_{rand}	λ/λ_{rand}
male	2.39 ± 0.18 (SD)	1.14 ± 0.04 (SD)	1.70 ± 0.13 (SD)	1.03 ± 0.02 (SD)	1.61 ± 0.12 (SD)	1.02 ± 0.02 (SD)
female	2.56 ± 0.18 (SD)	1.16 ± 0.03 (SD)	1.87 ± 0.16 (SD)	1.05 ± 0.03 (SD)	1.75 ± 0.15 (SD)	1.04 ± 0.02 (SD)
